# Alterations in the Gut Microbiota and Hepatitis-B-Virus Infection in Southern Chinese Patients With Coexisting Non-Alcoholic Fatty Liver Disease and Type-2 Diabetes Mellitus

**DOI:** 10.3389/fmed.2021.805029

**Published:** 2021-12-21

**Authors:** Weijia Han, Chunyang Huang, Yali Ji, Ling Zhou, Jinjun Chen, Jinlin Hou

**Affiliations:** ^1^Department of Liver Disease Center, Shenzhen Hospital, Southern Medical University, Shenzhen, China; ^2^Hepatology Unit, Department of Infectious Diseases, Nanfang Hospital, Southern Medical University, Guangzhou, China; ^3^Second Department of Liver Disease Center, Beijing Youan Hospital, Capital Medical University, Beijing, China; ^4^Hepatology Unit, Zengcheng Branch, Nanfang Hospital, Southern Medical University, Guangzhou, China; ^5^Chinese (Acute on) Chronic Liver Failure Consortium (Ch-CLIF.C), Shanghai, China

**Keywords:** HBV, NAFLD, microbiota, metabolome, hepatitis B virus

## Abstract

**Background:** Hepatitis B virus (HBV) infection has been reported to affect the bacterial characteristics in the host. We aimed to elucidate the compositional and functional characteristics of the microbiota in southern Chinese patients with coexistent HBV infection, non-alcoholic fatty liver disease (NAFLD), and type-2 diabetes mellitus (T2DM).

**Methods:** Healthy controls (HCs) and patients with coexistent NAFLD and T2DM were enrolled. Patients were divided into two groups: N1 (without HBV infection) and N2 (with HBV infection). Stool samples were collected for 16s RNA gene sequencing and untargeted metabolomics analysis.

**Results:** Bacterial diversity was decreased in the N2 group. There was a significantly lower abundance of bacteria of *Faecalibacterium, Gemmiger*, and *Clostridium_XIVA* genera, but a higher abundance of *Megamonas* and *Phascolarctobacterium* genera in the N2 group. Compared with the N1 group, the abundance of *Gemmiger* species was even lower, and alterations in the abundance of *Phascolarctobacterium* and *Clostridium_XIVA* genera only occurred in the N2 group. There were significantly different fecal metabolic features, which were enriched in glucose and lipid metabolic pathways (e.g., fatty acid and glycerophospholipid metabolism) between the N2 and HC groups. Metabolites in glycerophospholipid metabolism, such as Sn-3-o-(geranylgeranyl)glycerol1-phosphate, were even higher in the N2 group than in the N1 group. The decreased *Faecalibacterium* and *Gemmiger* contributed to the increased level of Sn-3-o-(geranylgeranyl) glycerol1-phosphate, palmitoylcarnitine, and serum triglycerides. *Clostridium_XIVA* species were positively correlated to 15(s)-hpete. *Megamonas* species were positively correlated with the serum level of glucose indirectly.

**Conclusions:** The distinct gut-microbiome profile associated with HBV infection has a role in lipid metabolism and glucose metabolism in patients with coexistent NAFLD and T2DM.

**Clinical Trial Registration:** www.ClinicalTrials.gov, identifier: NCT03525769.

## Introduction

Non-alcoholic fatty liver disease (NAFLD) is defined by fat accumulation in the liver. It is an important health issue worldwide. Gradually, NAFLD is becoming the most common cause of further progression to end-stage liver diseases (1). The increasing prevalence of NAFLD is related to type-2 diabetes mellitus (T2DM). Approximately 55% of patients with NAFLD also have T2DM (2). Patients with coexisting NAFLD and T2DM are 2–4-times more likely to develop end-stage liver diseases than those without T2DM (3). Excess glucose is the raw material for the synthesis of free fatty acids, which leads to increased levels of cholesterol and triglycerides (TG). Lipid accumulation due to increased glucose levels can cause liver inflammation in NAFLD (4, 5).

The term “microbiota” is a collective term for the microorganisms that live in or on the human body. “Microbiome” refers to the collection of genomes from all the microorganisms in an environment (e.g., gut). Microbiota dysbiosis is related to an increased serum level of TG and glucose tolerance in NAFLD and T2DM (6, 7). Several studies have summarized the altered composition of the microbiome in NAFLD (8), T2DM (9), and coexistence of NAFLD and T2DM (10). Foods (11) or drugs (12) that improve microbiota dysbiosis and alter microbiome composition may reduce plasma levels of glucose and TG in patients with NAFLD or T2DM.

The number of patients who have hepatitis B virus (HBV) infection, NAFLD, and T2DM is high (13). The prognosis of coexisting chronic hepatitis B (CHB) and NAFLD is worse than that of CHB alone or NAFLD alone (14, 15). Moreover, the HBV has been reported to affect bacterial characteristics in the host (16, 17). Hence, the compositional and functional characteristics of the microbiota in patients with coexisting HBV infection, NAFLD, and T2D merit exploration.

We recruited healthy people and patients with coexisting NAFLD and T2DM. The bacterial composition and functional characteristics of patients were analyzed through 16s RNA gene sequencing and untargeted metabolomics analysis. Our study provides ideas for future longitudinal studies to investigate the effect of the microbiota in the treatment of patients with coexistent HBV infection, NAFLD, and T2DM.

## Methods

### Ethical Approval of the Study Protocol

The study protocol was approved (NFEC-2018-023) by the Ethics Committee of Nanfang Hospital within Southern Medical University (Guangzhou, China) and registered at ClinicalTrials.gov (NCT04573283). All participants provided written informed consent for their data to be used in this study.

### Inclusion Criteria

The inclusion criteria for healthy controls (HCs) and patients were: (1) age ≥18 years; (2) men and women; (3) microecological agents (MAs) and antibiotics were not used; (4) excess alcohol was not consumed (alcohol intake <140 g/week for men; <70 g/week for women).

In addition to the four criteria mentioned above, two additional criteria were applicable for patients with coexisting NAFLD and T2DM (N1 group). The first criterion was that NAFLD was detected by transient elastography using FibroScan™ (Echosens, Paris, France). Liver biopsy is not acceptable for long-term dynamic monitoring of NAFLD stages. Therefore, non-invasive methods for NAFLD assessment have become essential. The controlled attenuation parameter (CAP) has demonstrated good accuracy for reflecting NAFLD severity (18, 19). In a recent study on gut microbiome (GM) signatures and the biological functions associated with liver fibrosis, Kwan et al. (20) used FibroScan to screen for NAFLD. In the present study, CAP >236 (dB/m) was diagnostic for NAFLD. The second criterion was that the T2DM diagnosis was assisted by the Endocrinology Department of Nanfang Hospital.

In addition to the criteria mentioned above, patients with coexisting HBV infection, NAFLD, and T2DM (N2 group) also had to meet one additional criteria: hepatitis B surface antigen (HBsAg) positivity with detectable HBV–DNA in serum, according to PCR.

### Exclusion Criteria

The exclusion criteria were people: (i) with symptoms of acute or chronic infection; (ii) with cancer; (iii) who were pregnant or lactating; (iv) using antibiotics or intestinal MAs within 2 weeks of enrollment.

### Patients

**Figure 1:**
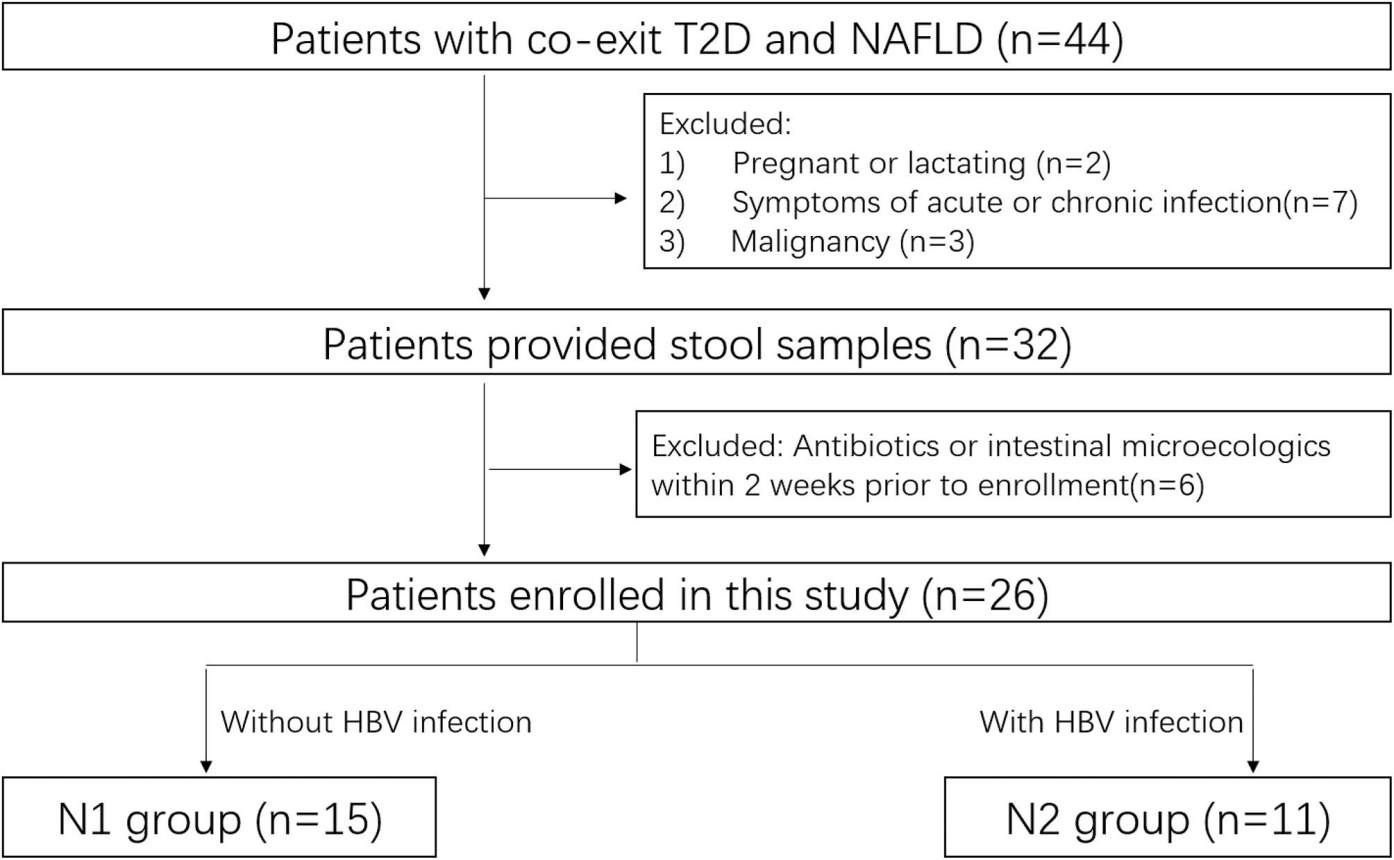
Flow diagram of patient enrollment.

This prospective observational study was carried out between October 2020 and December 2020 at Nanfang Hospital. HCs and patients with coexistent NAFLD and T2DM were enrolled. Patients underwent standard treatment for their particular disease regardless of their enrollment in this study (Figure 1).

Enrolled patients underwent sampling of stools and blood simultaneously. Levels of alanine aminotransferase (ALT), aspartate transaminase (AST), total bilirubin (TBIL), triglycerides (TG), total cholesterol (TC), and glucose were included as biochemical parameters in serum. Fecal samples were obtained in a plastic collection kit. All samples were stored at −80°C.

### 16s RNA Gene Sequencing in Stools

The DNA in the microbial community was extracted using the MagPure Stool DNA KF Kit B (Magen, Beijing, China) according to manufacturer instructions. DNA was quantified with a fluorometer in the Qubit® dsDNA BR Assay kit (Invitrogen, Carlsbad, CA, USA). DNA quality was checked by running aliquots on 1% agarose gel.

Variable region (V4) of the bacterial 16S rRNA gene was amplified with the degenerate PCR primers 515 forward (5′-GTGCCAGCMGCCGCGGTAA-3′) and 806 reverse (5′-GGACTACHVGGGTWTCTAAT-3′). Forward and reverse primers were tagged with an adapter, pad, and linker sequences from Illumina (San Diego, CA, USA). PCR enrichment was done in a reaction volume of 50 μl containing 30 ng of template, fusion PCR primer, and PCR master mix. PCR cycling conditions were 95°C for 3 min, 30 cycles of 95°C for 45 s, 56°C for 45 s, 72°C for 45 s, and final extension at 72°C for 10 min. PCR products were purified using Agencourt AMPure XP beads and eluted in elution buffer (Beckman Coulter, Fullerton, CA, USA). Libraries were qualified by a bioanalyzer (2,100 series; Agilent Technologies, Santa Clara, CA, USA). Validated libraries were used for sequencing on the HiSeq 2,500 platform, following the standard pipelines of Illumina, and generated 2 × 250 bp paired-end reads.

### Untargeted Metabolomics Analysis Using Stools

Stool samples were used for untargeted metabolomics analysis. A sample (100 mg) was weighed precisely and mixed with 800 μl of extract (methanol:acetonitrile:water = 2:2:1 (*v: v: v*), precooled at −20°C). Then, the sample was homogenized at 50 Hz for 5 min by a tissue lyser with two glass beads. Then, 10 min of ultrasound agitation in water at 4°C and for 1 h at −20°C was undertaken. After centrifugation at 25,000 rpm for 15 min at room temperature, the sample was mixed with 600 μl of complexation solution (methanol:H_2_O = 1:9 (*v:v*)), followed by vortex-mixing for 1 min, ultrasound agitation in water for 10 min at 4°C, and centrifugation at 25,000 rpm for 15 min at 4°C. Then, two-dimensional ultrahigh pressure liquid chromatography was done using a Waters system (Waltham, MA, USA). Tandem mass spectrometry was carried out using a Q Exactive™ high-resolution mass spectrometer (Thermo Fisher Scientific, Waltham, MA, USA) for the separation and detection of metabolites.

### Bioinformatics Analysis

Filtering and remaining high-quality clean data for analysis. Reads were spliced into tags. Selection of operational taxonomic units (OTUs) was achieved using a similarity cutoff of 97%. We clustered Tags into OTUs through USEARCH 7.0.1090. Species annotation was achieved by comparing OTUs with the Ribosomal Database Project (release date = 3 September 2021). Based on OTUs and annotation results, α-diversity and β-diversity were displayed. The Wilcoxon test was used to calculate α-diversity. The Wilcoxon rank-sum test was used to calculate β-diversity. The distinguished genus and predicted pathway were screened based on the Wilcoxon test (*p* < 0.05) and |log base 2-fold change (FC)| >1 through PICRUSt2 v2.2.0-b, R (v3.4.10). Linear discriminant analysis effect size (LEfSe) (https://huttenhower.sph.harvard.edu/galaxy/) was employed to explore different microbiota at different taxonomic levels (LEfSe is a software tool for finding higher-dimensional biomarkers and revealing genomic characteristics to assess whether differential microbiotas are expected.). The Spearman correlation coefficient revealed important patterns and relationships among dominant species. GMrepo (https://gmrepo.humangut.info/home) (8) was used to search the differential gut microbiota. At the class level, the microbiota with relative abundance <0.5% in all samples were merged into “Others.” The metabolome database of the Beijing Genomics Institute was used to analyze the key primary metabolites and intermediates of key metabolic pathways. The Human Metabolome Database (https://hmdb.ca/) was used to analyze human metabolites. The Lipidmaps (www.lipidmaps.org/resources/databases/index.php/) database was employed to identify the metabolites of lipids. Partial least squares-discriminant analysis (PLS-DA) was employed to analyze differences in metabolites between groups. The Kyoto Encyclopedia of Genes and Genomes (KEGG) database (www.genome.jp/) was used for the analysis of enrichment of the metabolic pathways of different metabolites.

### Statistical Analyses

Continuous variables are expressed as the mean ± SD. A *p* < 0.05 was considered significant. Statistical analyses were undertaken using SPSS 19 (IBM, Armonk, NY, USA). α-Diversity was evaluated using mothur-1.39.5/mothur and ggplot packages, and β-diversity was evaluated using QIIME1 and ggplot packages, within R 3.2.1 (R Institute for Statistical Computing, Vienna, Austria). Principal coordinates analysis (PCoA) was done using the mixOmics package within R.

## Results

### Characteristics of the Entire Cohort

The clinical characteristics of the entire cohort are shown in Table 1. There were no significant differences in age, height, weight, waist circumference, or heart rate between HCs and the N2 group (*p* > 0.05). However, the body mass index (BMI) of HCs (22.48 ± 1.52 kg/m^2^) was significantly lower than that of the N2 group (25.53 ± 2.54 kg/m^2^) (*p* < 0.05). There were no significant differences in age, height, weight, waist circumference, heart rate, BMI, CAP, or levels of ALT, AST, TBIL, TG, TC, or glucose between the N1 and N2 groups (*p* > 0.05).

**Table 1:** The baseline characteristic of N1 and N2 group.

	**Health (CON)**	**NAFLD and T2D (N1)**	**CHB accompanied by NAFLD and T2D (N2)**	P^*^	P^**^
n (male)	16(10)	15 (6)	11 (2)		
Age (year)	53.8 ± 9.84	47.67 ± 9.74	52.73 ± 6.6	0.150	0.757
Height (cm)	165.93 ± 8.81	162.87 ± 8.75	162.95 ± 8.25	0.980	0.391
Weight (kg)	62.2 ± 8.79	72.88 ± 11.45	68.15 ± 10.95	0.299	0.138
BMI	22.48 ± 1.52	27.48 ± 3.59	25.53 ± 2.54	0.138	**0.001**
Waistline (cm)		94.2 ± 8.59	91.55 ± 5.75	0.384	
Heart rate		74.67 ± 9.71	81.1 ± 12.84	0.167	
ALT (IU/L)		41.67 ± 33.95	36.52 ± 19.88	0.658	
AST (IU/L)		24.2 ± 12.83	27.97 ± 15.41	0.503	
TBIL (μmol/L)		28.95 ± 52.76	20.81 ± 19.06	0.631	
TG (mmol/L)		1.88 ± 1.22	2.45 ± 1.62	0.315	
TC (mmol/L)		5.44 ± 1.32	5.25 ± 1.11	0.704	
GLU (mmol/L)		9.11 ± 4.52	6.57 ± 1.09	0.082	
CAP (dB/m)	<236	315.23 ± 38.59	286.6 ± 36.53	0.068	

* *The comparison between N1 and N2 group;*

** *The comparison between N2 and CON group*.

*ALT, Alanine transaminase; AST, Aspartate transaminase; TBIL, Total bilirubin; TG, Triglyceride; TC, Total cholesterol; GLU, Glucose. The meaning of the bold values: p < 0.05*.

### Bacterial Characteristics of Patients With Coexistent HBV Infection, NAFLD, and T2DM

**Figure 2:**
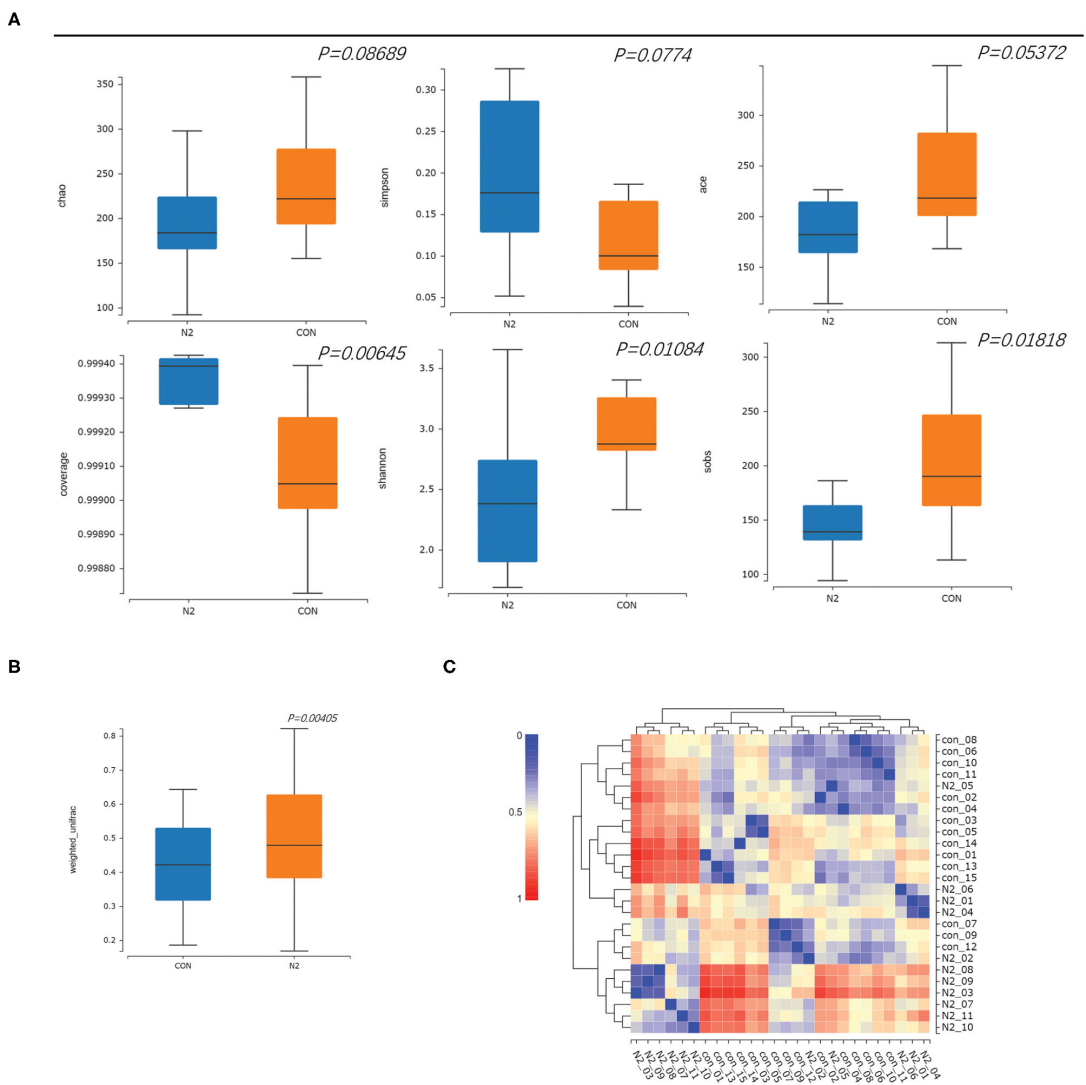
Comparison of α-diversity and β diversity in healthy controls (CON group) and patients with HBV infection (N2 group). **(A)** α-diversity and **(B)** β-diversity, in box plots; **(C)** β-diversity in a heatmap.

The saturated rarefaction curves based on the Shannon index and observed species indicated the high quality of sequences for all samples (Supplementary Figure 1). The richness and diversity of the HC and N2 groups were compared through the α-diversity metrics shown in Figure 1. There was no significant difference in the Chao index, Simpson index, or Ace index (*p* > 0.05). The coverage index of the N2 group was significantly higher than that of the HCs group (*p* < 0.05), whereas the Sobs index and the Shannon index of the N2 group were significantly lower than those of the HCs group (*p* < 0.05) (Figure 2A). The β-diversity of the N2 group was significantly lower than that of the HCs group (*p* < 0.05) (Figure 2B). The β-diversity heatmap showed higher indices between groups (Figure 2C). Significant differences in bacterial communities were analyzed through PCoA. The microbiota were separated in the first axis (PC1) (Supplementary Figure 2).

**Figure 3:**
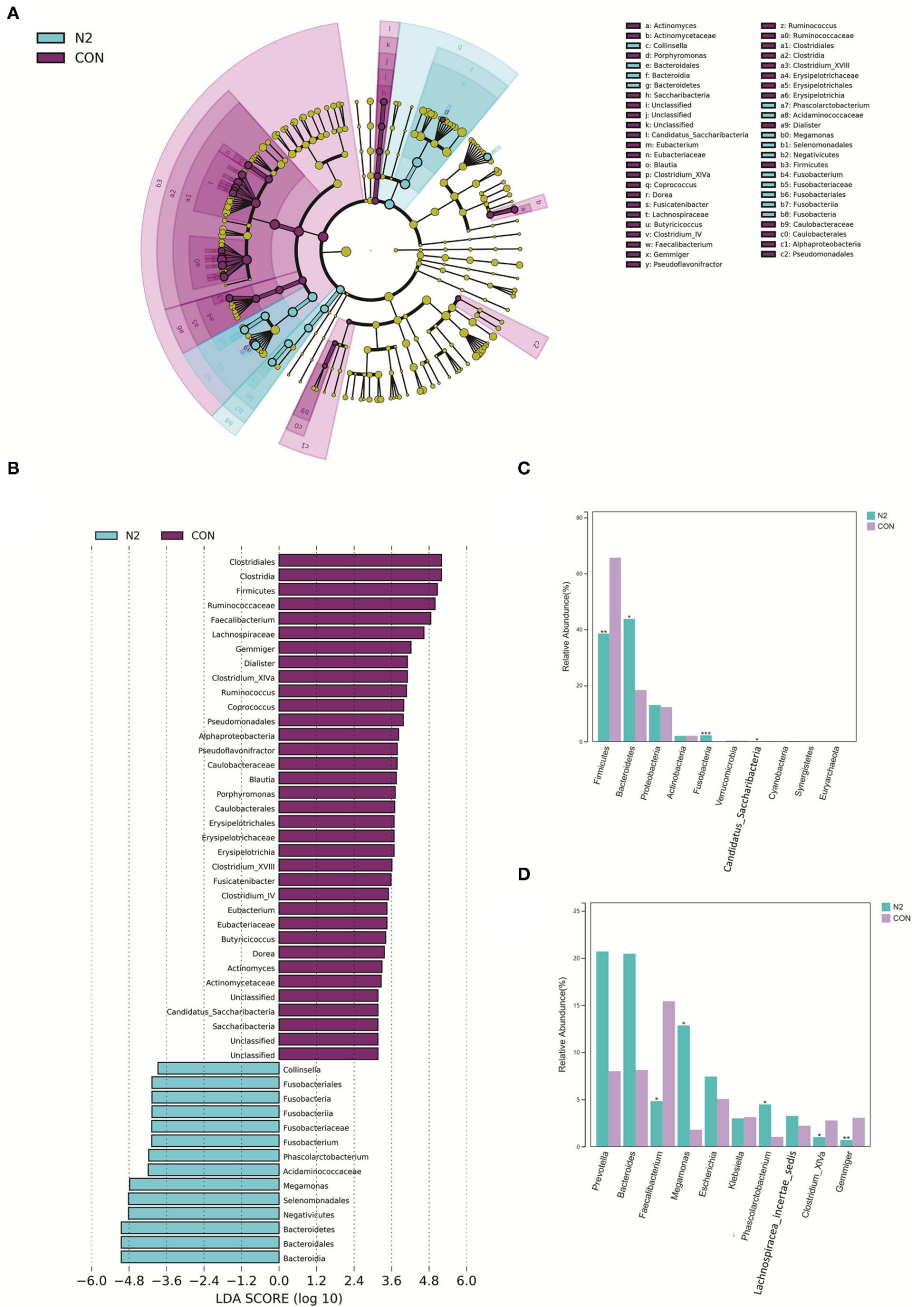
Bacterial composition in healthy controls (CON group) and patients with HBV infection (N2 group). **(A)** LEfSe cluster diagram of the N2 group. **(B)** LEfSe LDA of N2 and CON group. **(C)** Comparison of phyla taxa. **(D)** Comparison of genera taxa.

The bacterial composition of the N2 group was explored (Figure 2). At the phylum level, LEfSe clustering showed that microbiota of the HC group were concentrated in Firmicutes and Candidatus_Saccharibacteria, whereas those of the N2 group were concentrated in Bacteroidetes and Fusobacteria (Figures 3A,B). The Wilcoxon test showed that the abundance of Firmicutes was significantly higher in the HC group, whereas the abundance of Bacteroidetes and Fusobacteria was significantly higher in the N2 group (*p* < *0.05*) (Figure 3C). At the order level, the HC group was concentrated in Clostridiales, Caulobacterales, and Erysipelotrichales, and the N2 group was concentrated in Bacteroidales and Fusobacteriales (Figures 3A,B). At the genus level, there were 21 differentially expressed microbiota between the two groups (Supplementary Table 1). The Wilcoxon test showed that the abundance of *Faecalibacterium, Gemmiger*, and *Clostridium_XIVA* species was decreased significantly, whereas the abundance of *Megamonas* and *Phascolarctobacterium* species was increased significantly in the N2 group (*p* < *0.05*) (Figure 3D).

These data indicated a significant alteration of microbiota composition in patients with coexistent HBV infection, NAFLD, and T2DM. *Faecalibacterium, Gemmiger, Clostridium_XIVA, Megamonas*, and *Phascolarctobacterium* were the distinct genera in the GM.

### HBV Infection Affects the Bacterial Characteristics of Patients With Coexistent NAFLD and T2DM

**Figure 4:**
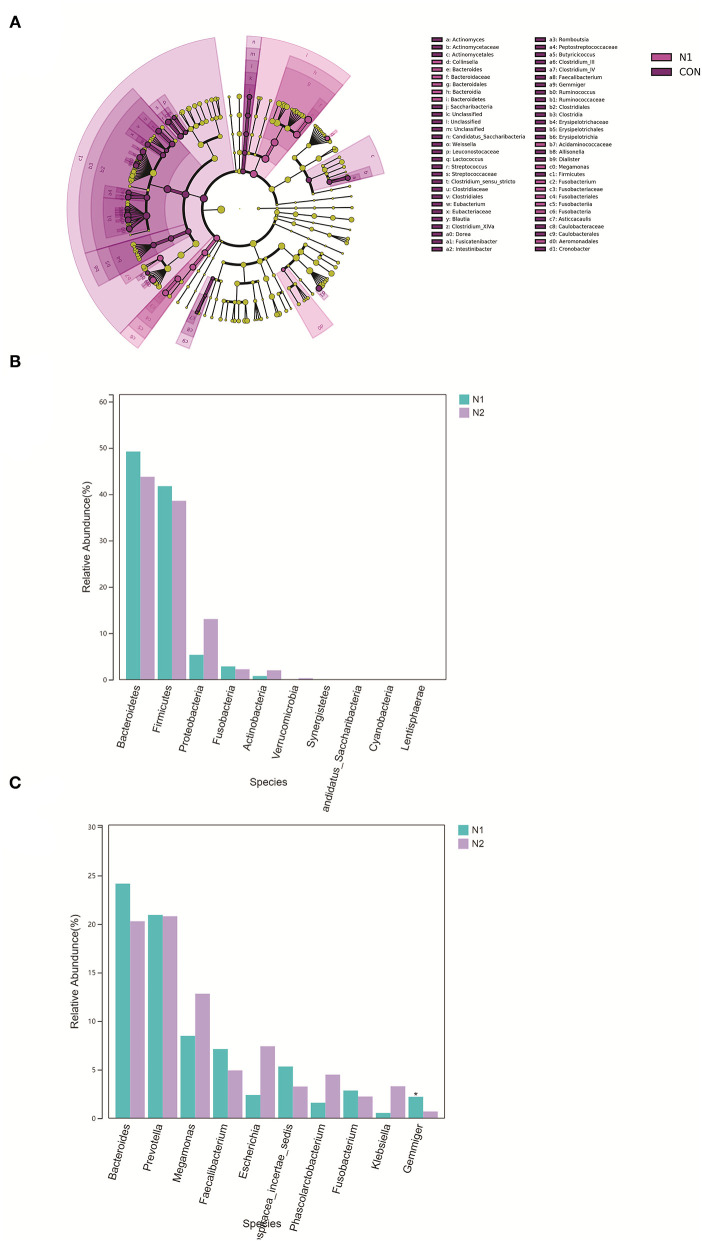
Bacterial composition in patients with HBV infection (N2 group) and patients without HBV infection (N1 group). **(A)** LEfSe cluster diagram of N1. **(B)** Comparison of the phyla taxa. **(C)** Comparison of the phyla taxa.

α-Diversity and β-diversity do not show a significant difference between the N1 group and N2 group (*p* > 0.05) (Supplementary Figures 3A–C). The microbiota were not separated in the axes PC1 and PC2 in the PCoA plot (Supplementary Figure 4). The microbiota of the N1 group were concentrated mainly in the phyla Bacteroidetes and Fusobacteria (Figure 4A). There were no significant differences in phyla between the N1 group and the N2 group (*p* > 0.05) (Figure 4B). There were 24 differentially expressed genera in the N1 group compared with those in the HC group (Supplementary Table 2). The abundance of *Gemmiger* species was lower in the N2 group than that in the N1 group (Figure 4C). A Venn diagram was used to compare the differentially expressed genera of the N1 group and the N2 group. An altered and distinct profile of *Faecalibacterium, Megamonas*, and *Gemmiger* species was observed in the N1 group and N2 group, whereas an altered profile of *Phascolarctobacterium* and *Clostridium_XIVA* species occurred only in the N2 group (Supplementary Figure 5A). These results indicated that HBV infection affected the bacterial characteristics of patients with coexistent NAFLD and T2DM.

The projects in the GMrepo database also suggested decreased abundance of *Faecalibacterium* species and increased abundance of *Megamonas* species in HBV infection, NAFLD, and T2DM projects. Expression of *Gemmiger* species was higher in the T2DM project. Alteration of *Clostridium* species was more pronounced in HBV-infection projects (Supplementary Figure 5B). These projects in datasets also supported the view that HBV infection affected the bacterial characteristics of patients with coexistent NAFLD and T2DM.

### Metabolic Characteristics of Patients With Coexistent HBV Infection, NAFLD, and T2DM

**Figure 5:**
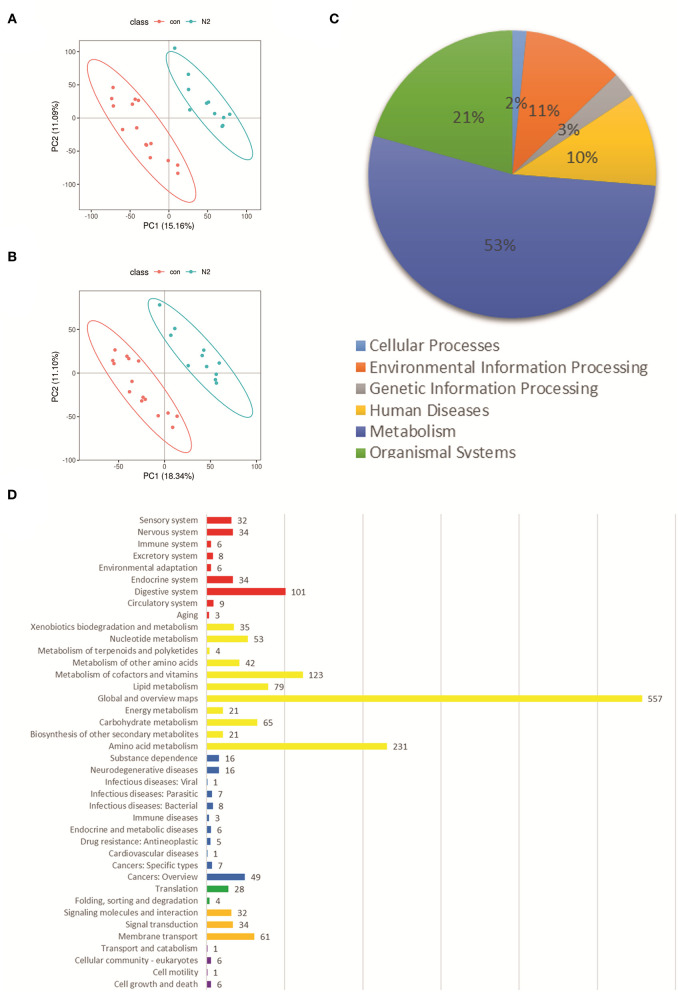
Fecal metabolic profiles in the N2 group and CON group. **(A)** PLS-DA of the two groups in ESI+ mode. **(B)** PLS-DA of the two groups in ESI– mode. **(C)** Classification of significantly altered metabolic features. **(D)** Enriched pathways of altered metabolic features using the KEGG database; Red represents “Organismal Systems;” Yellow represents “Metabolism;” green represents “Genetic Information Processing;” blue represents “Human Diseases;” Orange represents “Environmental Information Processing;” purple represents “Cellular Processes.” ESI, electrospray ionization.

Liquid chromatography–tandem mass spectrometry was used to evaluate metabolic profiles. PLS-DA showed apparent separation between the N2 group and HC group in electrospray ionization (ESI)+ and ESI– modes (Figures 5A,B). Heatmaps also revealed apparent separation between the N2 group and HC group in ESI+ and ESI– modes (Supplementary Figures 6, 7). A total of 768 and 1,743 features in ESI– and ESI+ modes were altered significantly between the N2 group and HC group according to the selection criteria of variable importance in projection (VIP) >1, |log_2_ FC|>1.2, and *p* < 0.05. Most of the metabolites belonged to metabolism and organismal systems (Figure 5C, Supplementary Table 3). Enrichment in glucose and lipid metabolic pathways was documented (Figure 5D). The metabolites in carbohydrates, carbonyl compounds, fatty acyls and derivatives, and sterol lipid family showed significant differential expression in the N2 group, including decreased expression of N-acetylmuramic acid, 4-hydroxybenzaldehyde, *Cis, cis*-muconic acid, 15(s)-hpete, 13(s)-hotre, 13(s)-hpotre, paromomycin, l-kynurenine, and 9-oxo-ode, and increased expression of Sn-3-o-(geranylgeranyl)glycerol 1-phosphate, (3α,7α,12α)-3,7,12,26-tetrahydroxycholestan-27-yl hydrogen sulfate, palmitoylcarnitine, estrone, desoxycortone, and cholate (Supplementary Tables 3–6). Palmitoylcarnitine is the metabolite of a fatty-acid metabolic pathway. An increase in the expression of palmitoylcarnitine indicated an increased level of fatty acids (Supplementary Figures 8, 9). The 15(s)-hpete is a metabolite of the arachidonic-acid metabolic pathway. A decrease in the expression of 15(s)-hpete indicated a reduced level of arachidonic acid (Supplementary Figure 10). Sn-3-o-(geranylgeranyl)glycerol 1-phosphate is a metabolite of the glycerophospholipid metabolic pathway. An increase in the expression of Sn-3-o-(geranylgeranyl)glycerol 1-phosphate indicated an increased level of glycerophospholipids (Supplementary Figure 11).

### HBV Infection Affects the Metabolic Characteristics of Patients With Coexistent NAFLD and T2DM

The PLS-DA showed an apparent separation between the N2 group and N1 group in ESI+ and ESI– modes (Supplementary Figures 12A,B). Analyses of enrichment of pathways using the KEGG database also showed a significant difference in the metabolic pathways between the two groups in ESI+ and ESI– modes (Supplementary Figures 12C,D). A total of 391 and 830 features in ESI– and ESI+ modes were altered significantly between the N2 group and N1 group (Supplementary Tables 7, 8). Compared with the N1 group, expression of Sn-3-o-(geranylgeranyl)glycerol 1-phosphate and (3α,7α,12α)-3,7,12,26-tetrahydroxycholestan-27-yl hydrogen sulfate was upregulated in the N2 group according to VIP>1, |log_2_ FC|>1.2, and *p* < 0.05 (Supplementary Table 9). These results indicated that HBV infection affected the metabolic characteristics of patients with coexistent NAFLD and T2DM.

### Association Between the Distinct GM Profile and Metabolites in Patients With Coexistent HBV Infection, NAFLD, and T2DM

**Figure 6:**
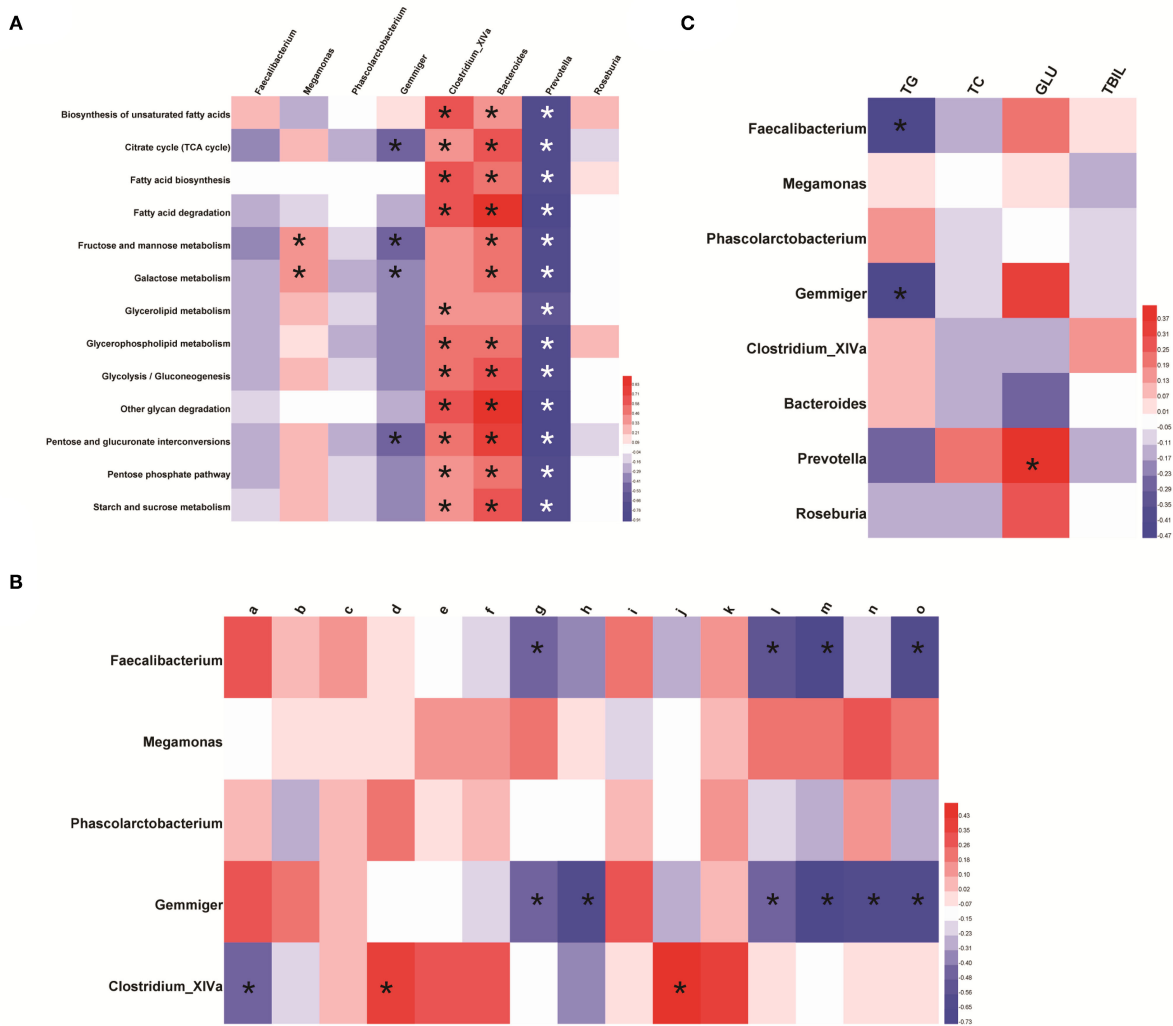
Association between the distinct gut-microbiome profile and metabolism. **(A)** Featured bacterial genera and pathways related to glucose metabolism and lipid metabolism. The predicted pathway was screened based on the Wilcoxon test (*p* < 0.05 and |log_2_ FC| >1). **(B)** Association between the distinct gut-microbiome profile and differential metabolites related to glucose metabolism and lipid metabolism. Here, a represents N-acetylmuramic acid; b represents 4-hydroxybenzaldehyde; c represents *Cis, cis*-muconic acid; d represents 15(s)-hpete; e represents 13(s)-hotre; f represents 13(s)-hpotre; g represents Sn-3-o-(geranylgeranyl)glycerol 1-phosphate; h represents (3α,7α,12α)-3,7,12,26-tetrahydroxycholestan-27-yl hydrogen sulfate; i represents paromomycin; j represents L-kynurenine; k represents 9-oxo-ode; l represents palmitoylcarnitine; m represents estrone; *n* represents desoxycortone; o represents cholate. **(C)** Featured bacterial genera and clinical manifestations related to glucose metabolism and lipid metabolism. *p < 0.05. Red represents a positive correlation and, the darker the color, the more significant is the correlation; blue represents a negative correlation and, the darker the color, the stronger is the correlation.

Enrichment of functional pathways was predicted by Picrust. Glucose- and lipid metabolism-related pathways in KEGG level three were compared with the distinct GM profile through the Spearman correlation analysis. We noted integration between the distinct GM profile and lipid metabolic pathways and the glucose metabolic pathways. *Megamonas* species were significantly positively correlated with fructose and mannose metabolic pathways and galactose metabolism (*p* < 0.05). *Gemmiger* species were significantly negatively correlated with the tricarboxylic acid (TCA) cycle, pentose and glucuronate interconversions, fructose and mannose metabolic pathways, and galactose metabolism (*p* < 0.05). *Clostridium_XIVA* species were significantly positively correlated with several pathways of glucose and lipid metabolism, including the TCA cycle (Figure 6A and Supplementary Table 10).

The Spearman correlation analysis showed integration between the distinct GM profile and metabolites in lipid and glucose metabolic pathways. *Clostridium_XlVa* species were positively correlated with 15(s)-hpete and l-kynurenine, and negatively correlated with N-acetylmuramic acid. Sn-3-o-(geranylgeranyl)glycerol 1-phosphate, palmitoylcarnitine, estrone, and cholate were negatively correlated with *Faecalibacterium* and *Gemmiger* species. (3α,7α,12α)-3,7,12,26-tetrahydroxycholestan-27-yl hydrogen sulfate and desoxycortone were negatively correlated with *Gemmiger* species (Figure 6B and Supplementary Table 11).

The distinct GM profile was significantly correlated with levels of TG, TC, and glucose. An increased abundance of *Faecalibacterium* and *Gemmiger* species was negatively correlated with the TG level (*p* < 0.05). *Prevotella* species, whose abundance was increased indirectly through *Megamonas* species, were positively correlated with the glucose level (Figure 6C and Supplementary Table 12).

## Discussion

Hepatitis B virus has been reported to affect the bacterial characteristics of the host. The composition of the microbiota in patients with HBV infection may not be identical to that of people not infected with the HBV. We focused, for the first time, on the composition and functional features of the GM and chronic HBV infection in patients with coexistent NAFLD and T2DM. In this way, we aimed to provide new insights into host–GM interactions in this population.

The diversity and richness of bacteria in the gut are important for maintaining intestinal homeostasis, the intestinal mucosal barrier, the immune system, and metabolism of the host. Reduced diversity is a characteristic of microbiota dysbiosis (21–23). In this work, α-diversity and β-diversity were reduced significantly in patients with coexistent HBV infection, NAFLD, and T2DM. Typically, pathobionts (organisms that can cause harm under certain circumstances) are maintained at low levels within the healthy gut, but changes in the composition may lead to an increased number of pathogenic bacteria (24, 25). Some studies have suggested that the increased abundance of bacteria of the phylum *Firmicutes*, a decreased abundance of bacteria of the phylum *Bacteroidetes*, and an increased *Firmicutes*/*Bacteroidetes* ratio are associated with NAFLD prognosis (26, 27). However, some researchers have suggested otherwise. They have found that Firmicutes continue to consist of seven probiotic classes [including many types of lactic-acid probiotics (28)], whereas bacteria belonging to *Bacteroidetes* and *Fusobacteria* genera are increased in many chronic diseases (16, 17). Therefore, compositional changes at the genus level are important (27). We found that the bacterial composition of patients with coexistent HBV infection, NAFLD, and T2DM was concentrated in the *Bacteroidetes* and *Fusobacteri*a phylum, whereas the microbiota of health were concentrated in *Firmicutes* phylum. Twenty-one genera showed differential expression in those patients. *Faecalibacterium, Gemmiger*, and *Clostridium_XIVA* genera showed decreased abundance and *Megamonas* and *Phascolarctobacterium* showed an increased abundance in a distinct GM profile. Zeng et al. also suggested that HBV infection led to a decreased abundance of bacteria of the genera *Faecalibacterium* and *Clostridium* (16).

Studies have found an association between HBV infection and reduction in bacterial diversity (16). Researchers have revealed an association between HBV infection and altered bacterial composition in chronic liver diseases (16, 17). Zhu and colleagues found that the bacterial diversity and total count of OTUs were changed significantly in mice infected with the HBV (29). In this study, compared with patients with coexistent HBV infection, NAFLD, and T2DM, there was no significant difference in the bacterial diversity in patients not infected with the HBV. In the latter, the bacterial composition was also focused on the *Bacteroidetes* and *Fusobacterium* phylum. At the genus level, altered abundance of *Phascolarctobacterium* and *Clostridium_XIVA* occurred only in patients infected with the HBV. Furthermore, a decreased abundance of *Gemmiger* species was more obvious in patients with HBV infection than in those without HBV infection. These data indicated that HBV infection affected the bacterial characteristics of patients with coexistent NAFLD and T2DM. Wu et al. (8) established the GMrepo database, which enables the exploration of the bacterial characteristics of common diseases at the genus level. Projects in that database also suggested that HBV infection affected the distinct GM profile.

Researchers have referred to the HBV as a “metabolovirus.” A recent study suggested that viruses may “reprogram” the host metabolism toward a more lipogenic and adipogenic status (30). HBV X upregulates the expression of fatty acid-binding protein and plays a central part in glucose and *de novo* lipid synthesis (31). The HBV is thought to be a superposition of hepatic steatosis, glucose metabolism, and hepatic lipid-accumulation pathways (32, 33). Therefore, the metabolites and metabolic characteristics of patients with coexistent HBV infection, NAFLD, and T2DM must be explored. Through metabolomics analysis, we found that the metabolites in carbohydrates, carbonyl compounds, fatty acyls and derivatives, and sterol lipids family were altered significantly in patients with coexistent HBV infection, NAFLD, and T2DM. Compared with people not infected with the HBV, those with HBV infection had a higher level of metabolites related to metabolic pathways, such as Sn-3-o-(geranylgeranyl)glycerol 1-phosphate.

We also revealed an integrated “cross-omics” analysis to better understand the link between the GM and metabolome in patients with coexistent HBV infection, NAFLD, and T2DM. Studies have suggested that bacteria of *Gemmiger* and *Faecalibacterium* genera affect metabolites significantly and are involved in the metabolism of fatty acids and glycerophospholipids (34). In this study, *Faecalibacterium, Megamonas*, and *Phascolarctobacterium* species facilitated carbohydrate-fermentation pathways, including the TCA cycle. *Gemmiger* species were negatively related to glucose metabolism, including the TCA cycle and galactose metabolism. Short-chain fatty acids (SCFAs) reduce lipid accumulation and insulin resistance (35). SCFAs-producing microbiotas are involved in gluconeogenesis, reduce the TG level, and promote energy storage (36). Studies have suggested that the SCFAs-producing microbiotas are reduced in NAFLD (37), T2DM (38), and HBV infection (39). Similarly, the distinct GM profile in patients with coexistent HBV infection, NAFLD, and T2DM in our study, including *Faecalibacterium, Megamonas*, and *Phascolarctobacterium* genera involved SCFA production, whereas *Gemmiger* species did not (40–44).

Studies have suggested that the expression of palmitoylcarnitine (product of fatty-acid metabolism) was upregulated along with an increased abundance of *Faecalibacterium* species (45). We found that palmitoylcarnitine in the fatty-acid metabolic pathway and Sn-3-o-(geranylgeranyl)glycerol 1-phosphate in the glycerophospholipid metabolic pathway were negatively correlated to *Faecalibacterium* and *Gemmiger* genera. Studies have suggested that the N-terminal domain of *Clostridium perfringens* alpha-toxin shows sequence and predicted structural homologies with the N-terminal region of arachidonate 5-lipoxygenase (46). We found that 15(s)-hpete in the arachidonic-acid metabolic pathway was positively correlated with *Clostridium_XIVA* species.

Patients with NAFLD with HBV infection have been shown to have significantly higher serum levels of fasting glucose, TG, and TC than those NAFLD patients not suffering from HBV infection (47). CHB is followed by increased levels of fatty acids, hepatic glucose production, and impaired glucose tolerance (48, 49). The clinical indicators in the present study also showed a negative relationship between the TG level and *Faecalibacterium* and *Gemmiger* genera. The abundance of *Prevotella* species was increased indirectly through *Megamonas* species (Supplementary Figure 13 and Supplementary Table 13) and was positively correlated with the glucose level.

The main limitation of our study was the relatively small cohort. However, based on projects in the GMrepo database and previous studies, we found a strong relationship between the altered bacterial composition and HBV infection in patients with coexistent NAFLD and T2DM.

## Conclusions

We revealed an association between altered bacterial characteristics and HBV infection in patients with coexistent NAFLD and T2DM. The distinct GM profile may have a functional role in glucose metabolism and lipid metabolism. Our study provides a basis for GM regulation as a potential therapeutic target for patients with coexistent HBV infection, NAFLD, and T2DM living in southern China.

## Data Availability Statement

The datasets presented in this study can be found in online repositories. The names of the repository/repositories and accession number(s) can be found at: https://www.ncbi.nlm.nih.gov/, BioProject ID: PRJNA780085.

## Ethics Statement

The studies involving human participants were reviewed and approved by Nanfang Hospital, Southern Medical University, Guangzhou, China (Approval No. NFEC-2018-023; ClinicalTrials.gov ID. NCT03525769). The patients/participants provided their written informed consent to participate in this study.

## Author Contributions

WH: performed the statistical analysis. CH and YJ: carried out the study and collected stools specimens. LZ: assistance for data acquisition and analysis. JC and JH: carried out literature research and manuscript editing. All authors contributed to the article and approved the submitted version.

## Funding

This article was supported by (1) Sanming Project of Medicine in Shenzhen (No. SZSM 201911001); (2) Research Foundation of Shenzhen Hospital of Southern Medical University (PY2021YM04); (3) National Natural Science Foundation of China (Grant No. 82000544); (4) Beijing iGandan Foundation (RGGJJ-2021-029); (5) Beijing Municipal Administration of Hospitals Incubating Program (Code: PX2019062); and (6) China Postdoctoral Science Foundation (2021M701612).

## Conflict of Interest

The authors declare that the research was conducted in the absence of any commercial or financial relationships that could be construed as a potential conflict of interest.

## Publisher's Note

All claims expressed in this article are solely those of the authors and do not necessarily represent those of their affiliated organizations, or those of the publisher, the editors and the reviewers. Any product that may be evaluated in this article, or claim that may be made by its manufacturer, is not guaranteed or endorsed by the publisher.

## Supplementary Material

Supplementary Figure 1 Rarefaction curves of all samples. **(A)** Shannon Index. **(B)** Observed species.

Supplementary Figure 2 PCoA revealing the bacterial communities between the N2 group and CON group.

Supplementary Figure 3 Comparison of α-diversity and β-diversity in patients with HBV infection (N2 group) and patients without HBV infection (N1 group). **(A)** α-diversity and **(B)** β-diversity in box plots. **(C)** β-diversity in a heatmap; ^*^Diversity coefficient >0.6.

Supplementary Figure 4 PCoA revealing the bacterial communities between the N1 group and N2 group.

Supplementary Figure 5 **(A)** Venn diagram of distinguished expressed genera in patients with HBV infection (N2 group) and those without HBV infection (N1 group). **(B)** Abundance of the distinct gut-microbiome profile in the GMrepo database.

Supplementary Figure 6 Heatmap of differential metabolites in ESI+ mode. Red represents a positive relationship; green represents a negative relationship. ESI, electrospray ionization.

Supplementary Figure 7 Heatmap of differential metabolites in ESI–mode. Red represents a positive relationship; green represents a negative relationship. ESI, electrospray ionization.

Supplementary Figure 8 Palmitoylcarnitine in fatty acid-degradation pathway.

Supplementary Figure 9 Palmitoylcarnitine in fatty acid-degradation metabolic pathway.

Supplementary Figure 10 15(s)-hpete in an arachidonic-acid metabolic pathway.

Supplementary Figure 11 Sn-3-o-(geranylgeranyl)glycerol 1-phosphate in a glycerophospholipid metabolic pathway.

Supplementary Figure 12 Fecal metabolic profiles in the N2 group and N1 group. **(A)** PLS-DA of the two groups in ESI+ mode. **(B)** PLS-DA of the two groups in ESI–mode. **(C)** Enriched pathways of altered metabolic features in ESI+ mode according to the KEGG database. **(D)** Enriched pathways of altered metabolic features in ESI– mode according to the KEGG database.

Supplementary Figure 13 The complicated network pattern between the distinct gut-microbiome profile. ^*^Correlation coefficient >0.5 or < −0.5.
