# Supplementary figures and images for: Alterations in the Gut Microbiota and Hepatitis-B-Virus Infection in Southern Chinese Patients With Coexisting Non-Alcoholic Fatty Liver Disease and Type-2 Diabetes Mellitus
Source: Front Med (Lausanne). 2021 Dec 21;8:805029. doi: 10.3389/fmed.2021.805029 (PMC8724037; doi:10.3389/fmed.2021.805029)

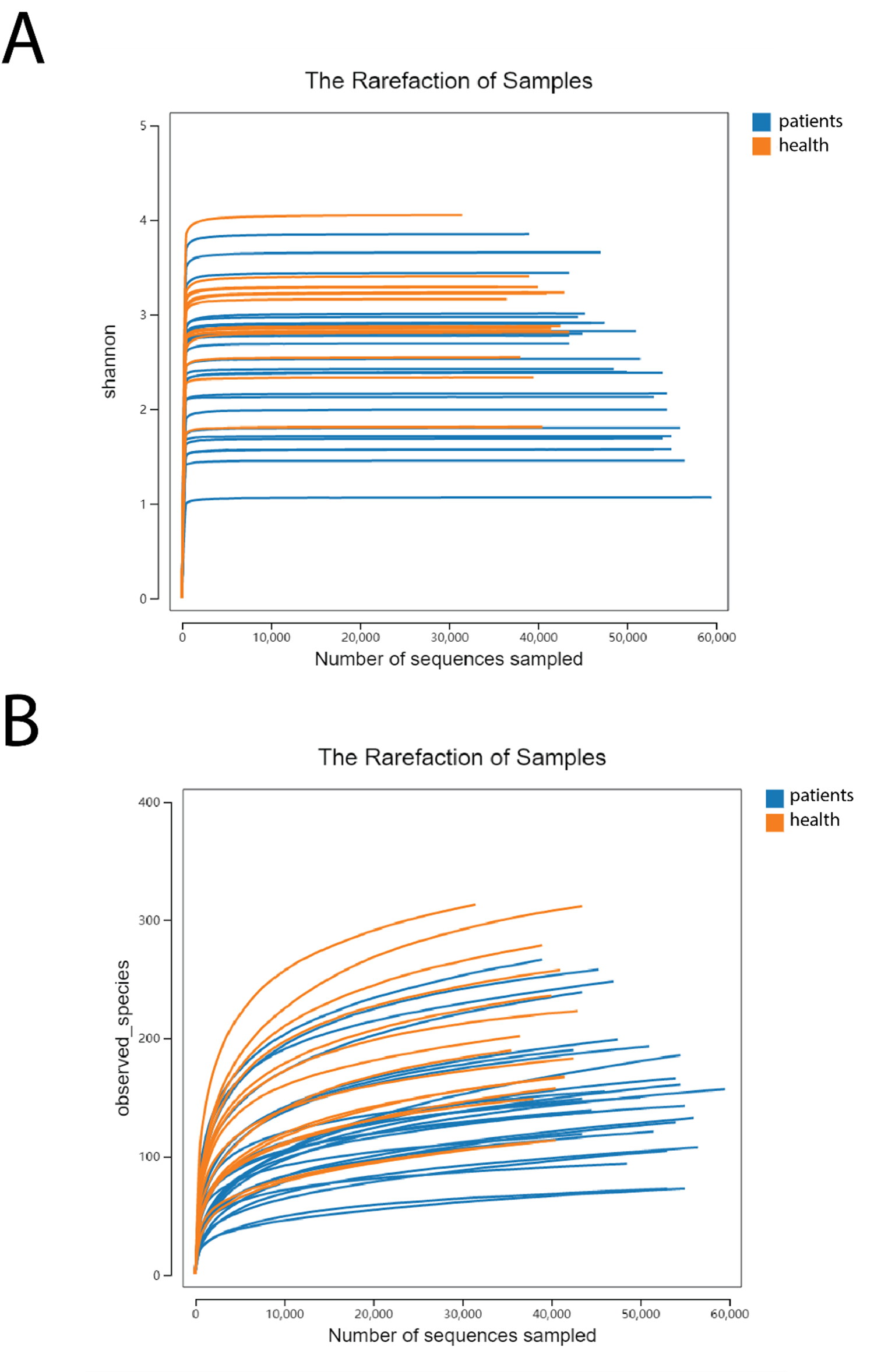

Supplement: Supplementary Figure 1 — Rarefaction curves of all samples. (A) Shannon Index. (B) Observed species. [file Image_1.PNG]

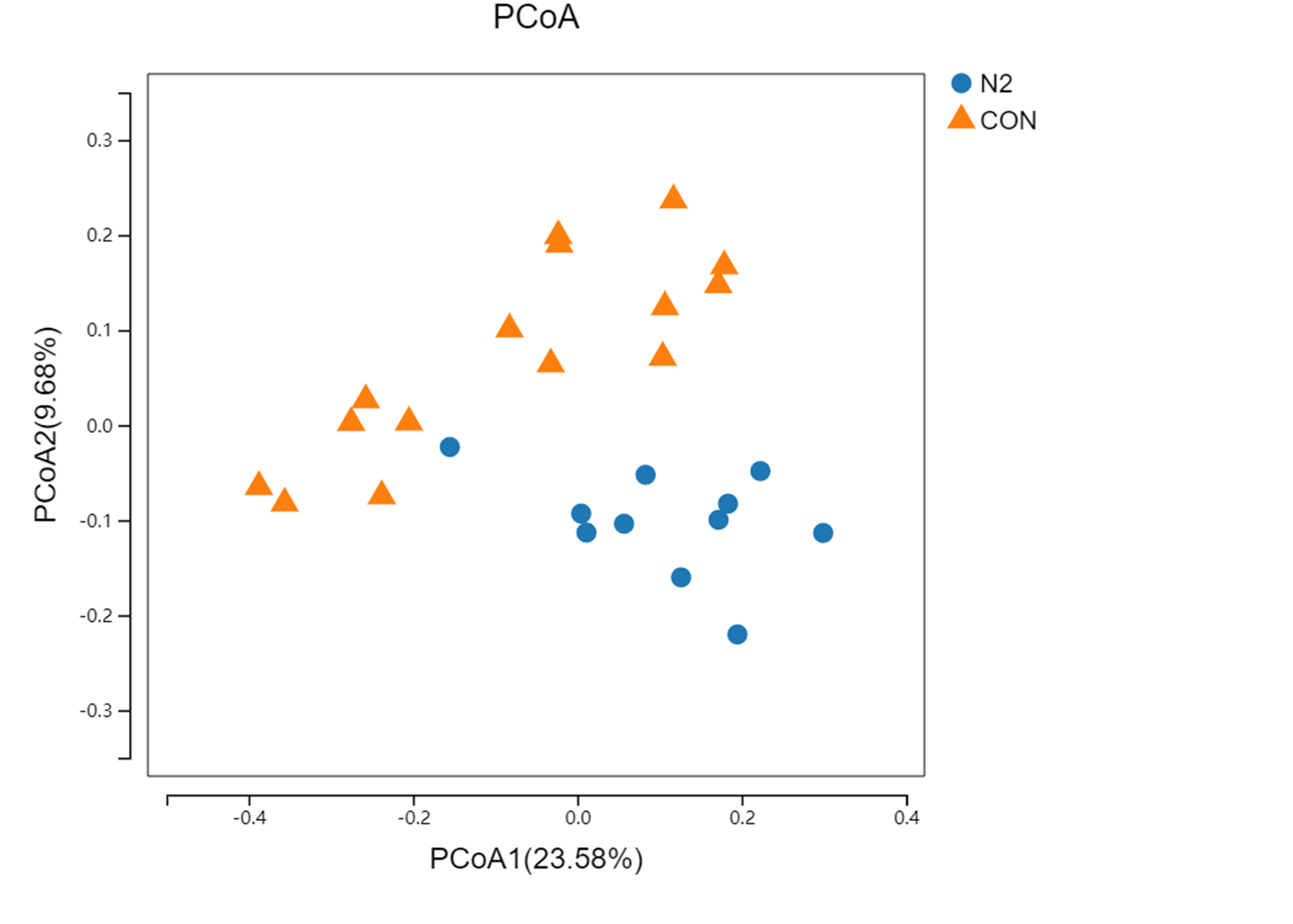

Supplement: Supplementary Figure 2 — PCoA revealing the bacterial communities between the N2 group and CON group. [file Image_2.PNG]

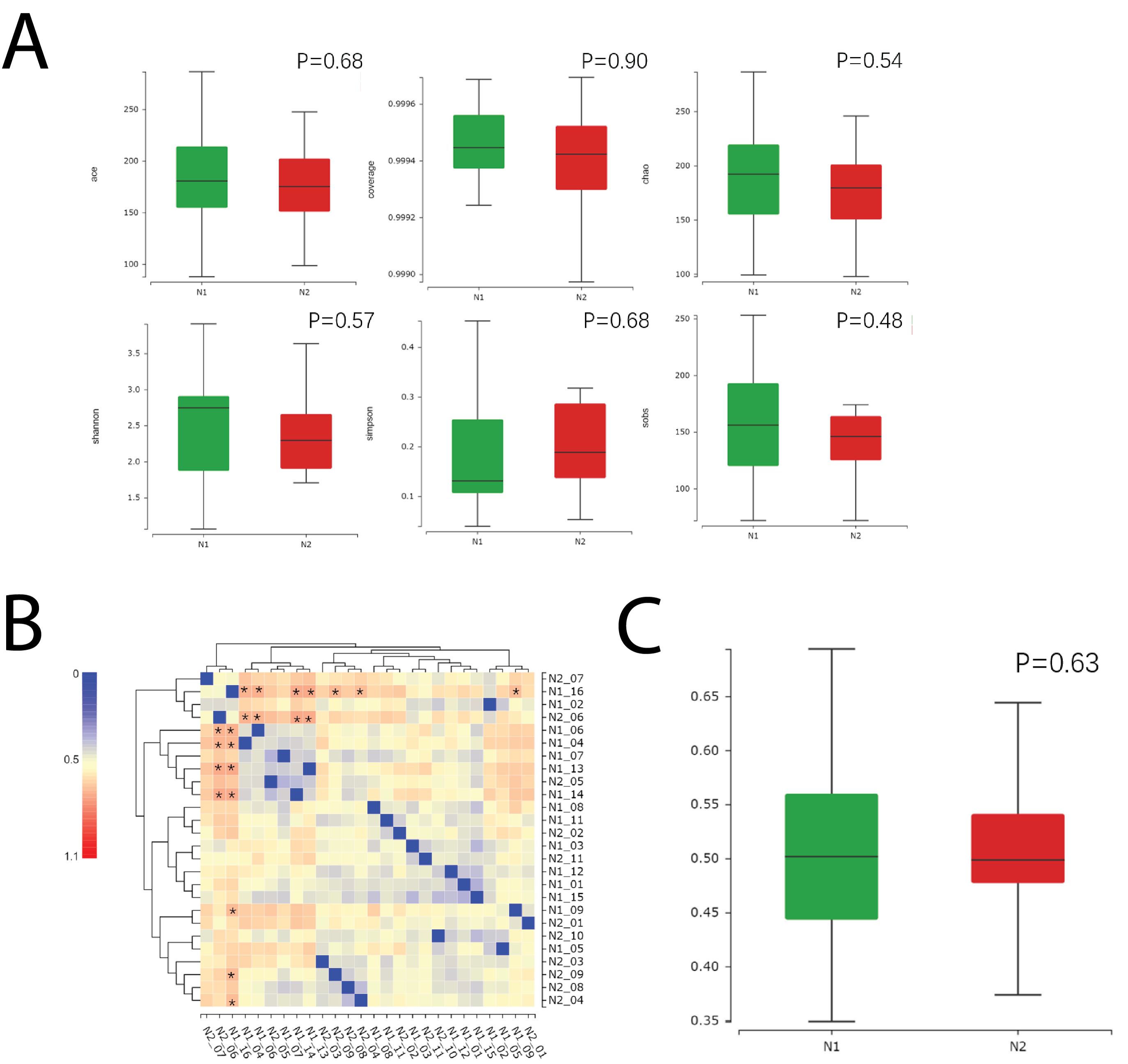

Supplement: Supplementary Figure 3 — Comparison of α-diversity and β-diversity in patients with HBV infection (N2 group) and patients without HBV infection (N1 group). (A) α-diversity and (B) β-diversity in box plots. (C) β-diversity in a heatmap; *Diversity coefficient >0.6. [file Image_3.PNG]

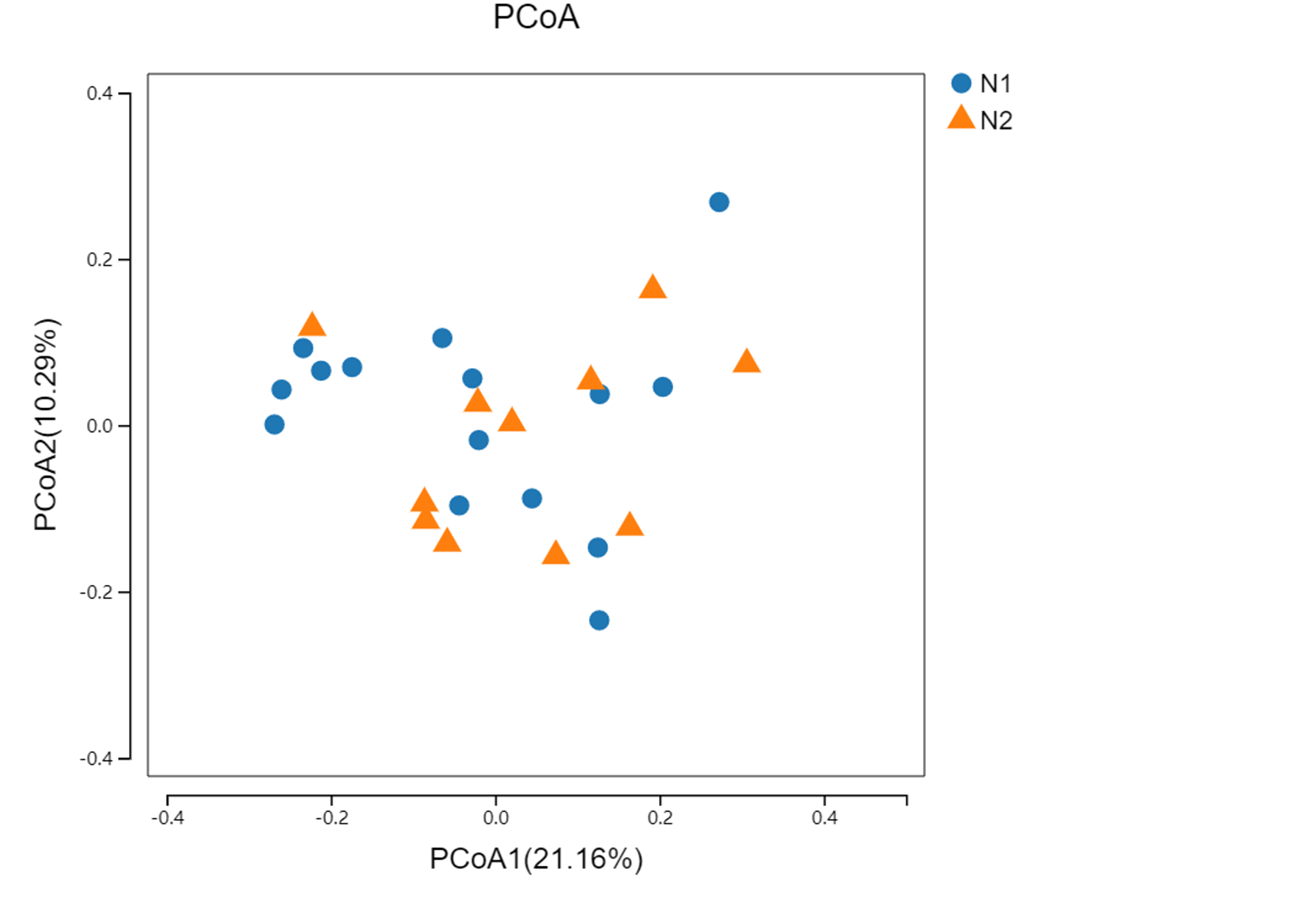

Supplement: Supplementary Figure 4 — PCoA revealing the bacterial communities between the N1 group and N2 group. [file Image_4.PNG]

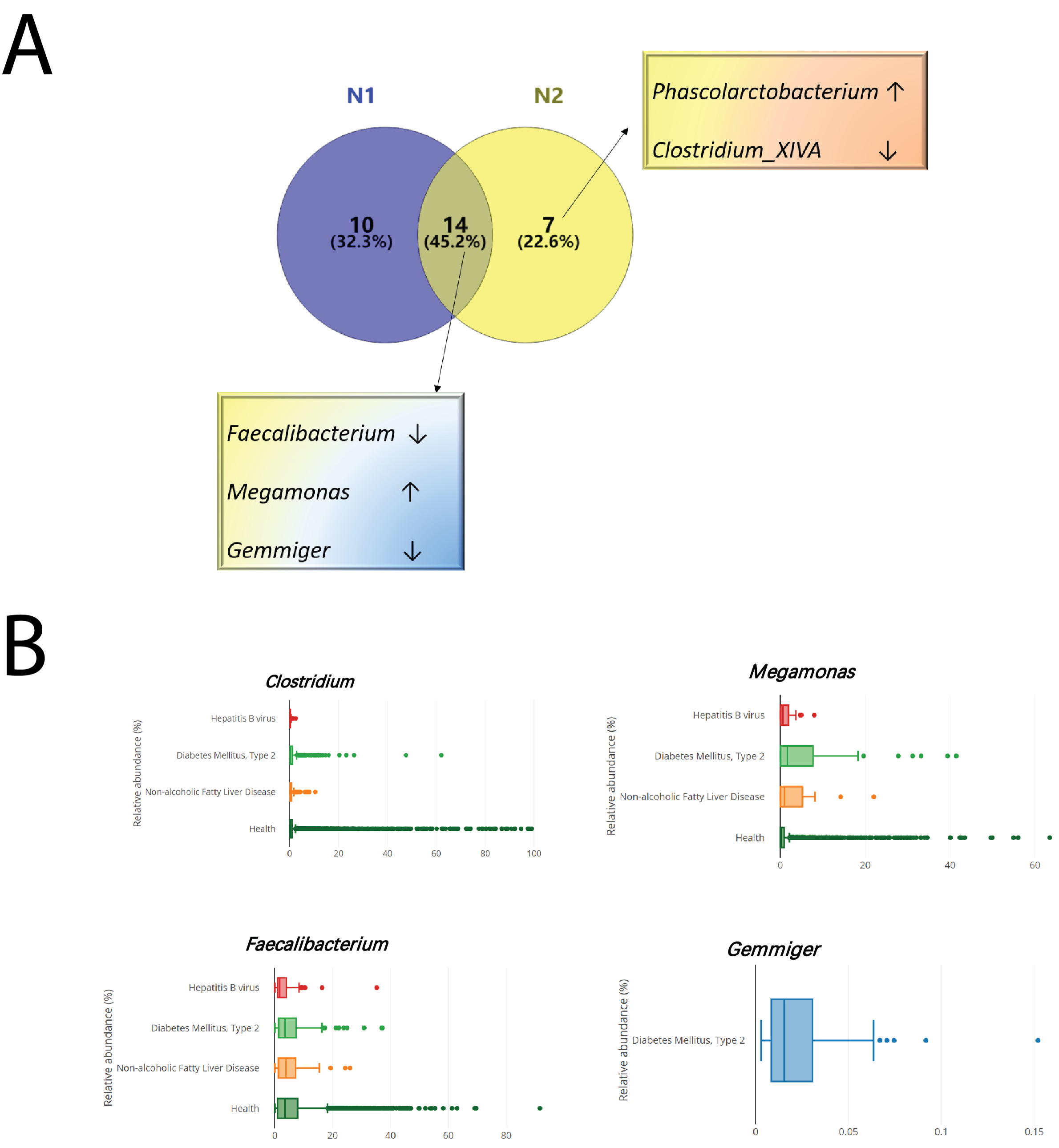

Supplement: Supplementary Figure 5 — (A) Venn diagram of distinguished expressed genera in patients with HBV infection (N2 group) and those without HBV infection (N1 group). (B) Abundance of the distinct gut-microbiome profile in the GMrepo database. [file Image_5.PNG]

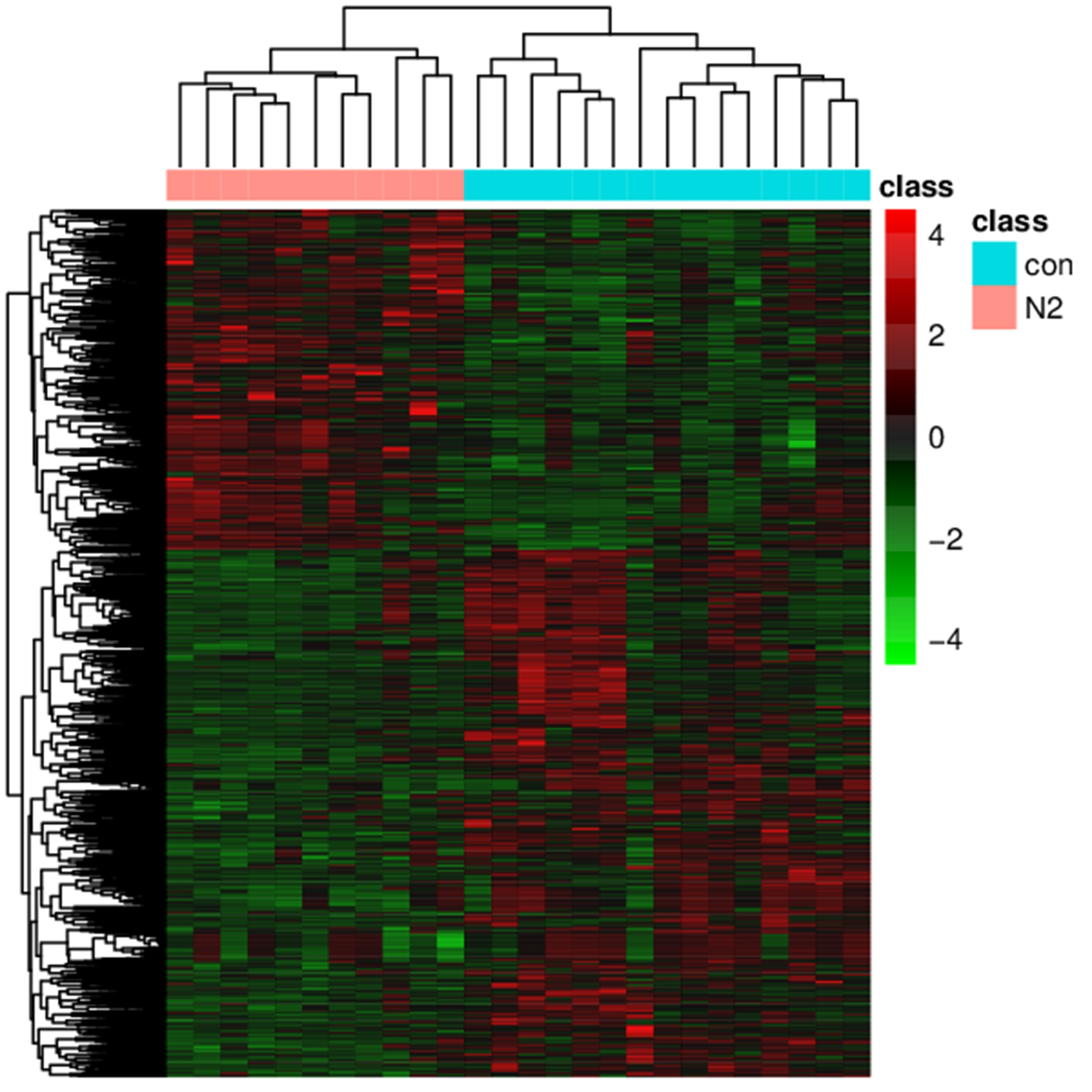

Supplement: Supplementary Figure 6 — Heatmap of differential metabolites in ESI+ mode. Red represents a positive relationship; green represents a negative relationship. ESI, electrospray ionization. [file Image_6.PNG]

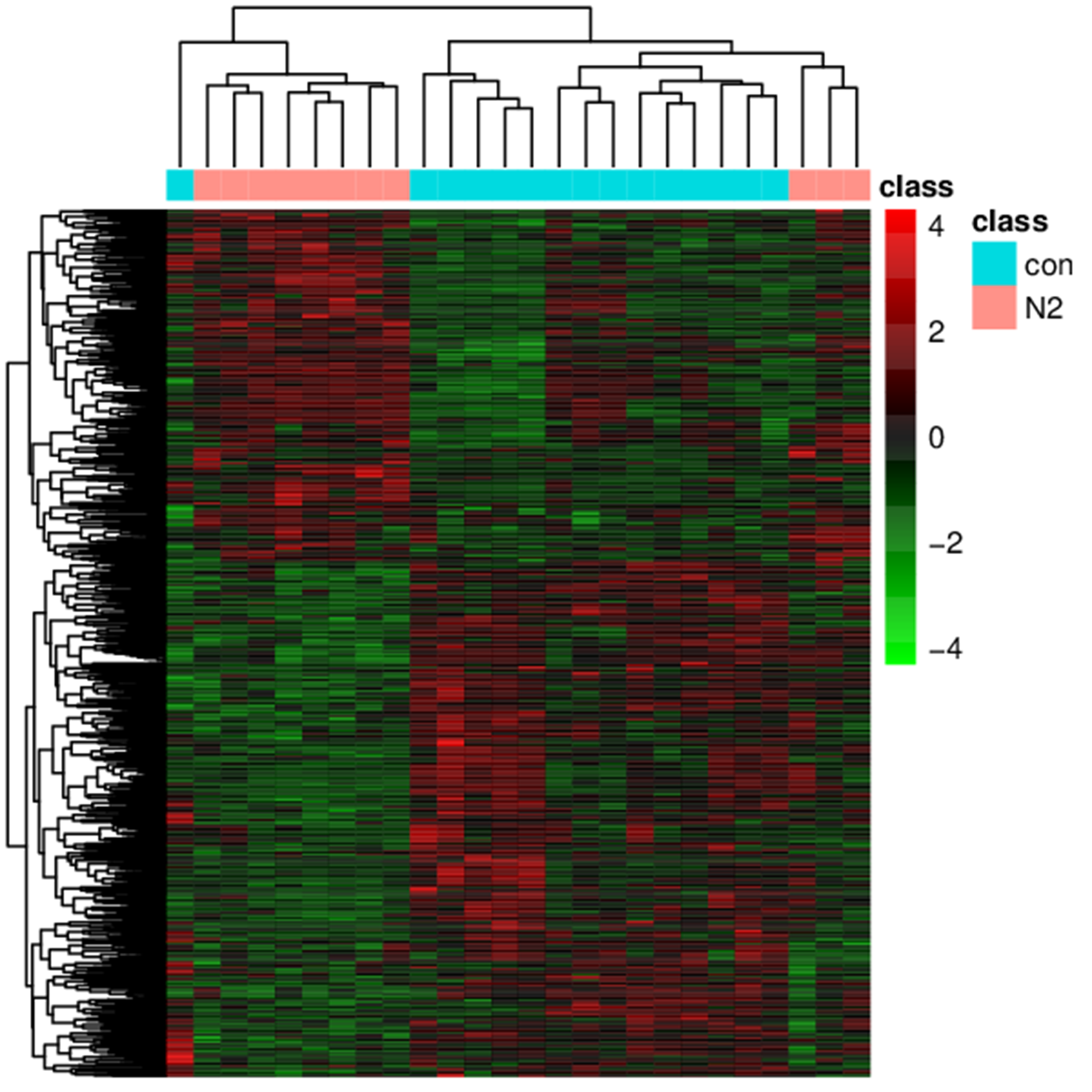

Supplement: Supplementary Figure 7 — Heatmap of differential metabolites in ESI–mode. Red represents a positive relationship; green represents a negative relationship. ESI, electrospray ionization. [file Image_7.PNG]

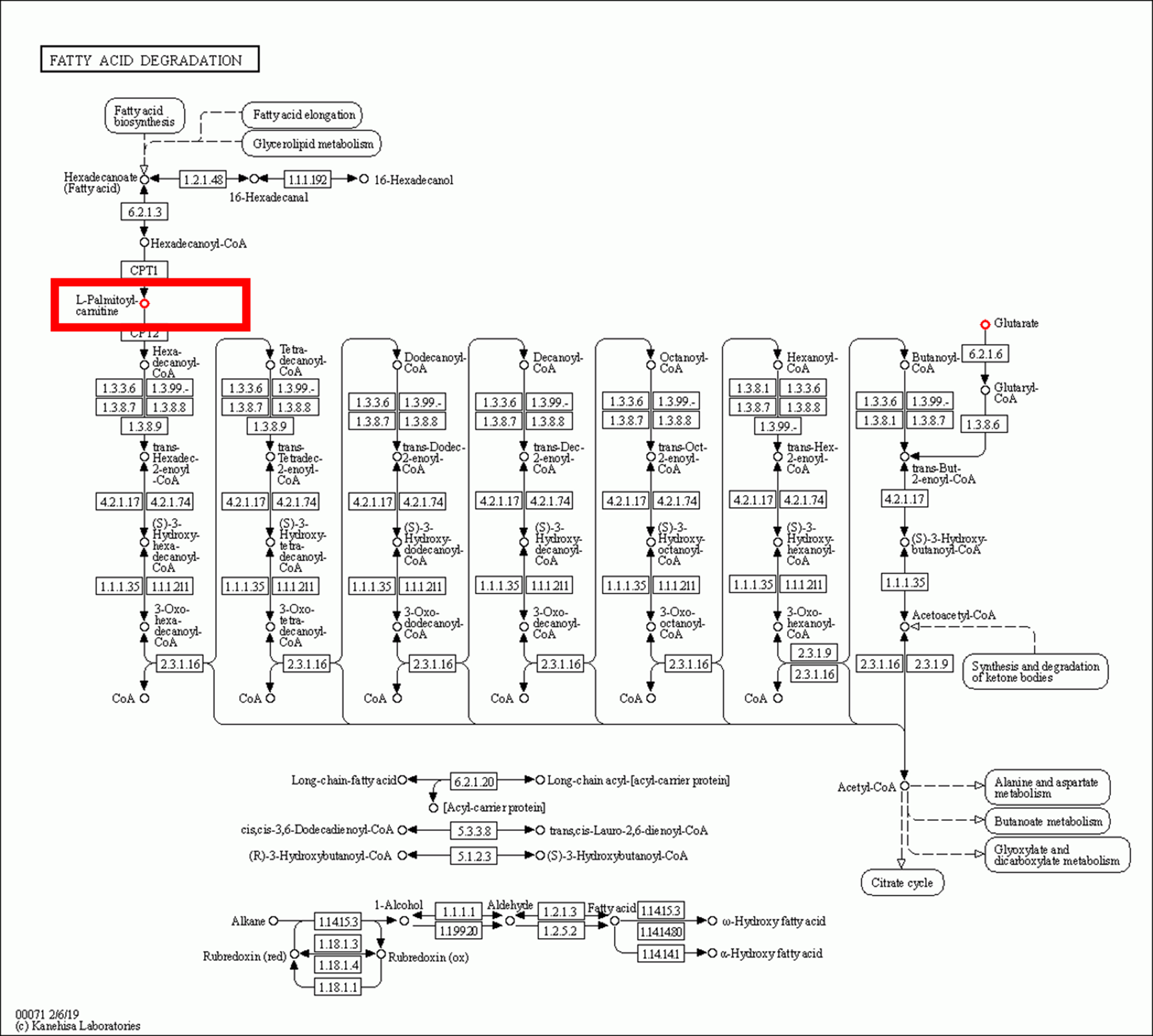

Supplement: Supplementary Figure 8 — Palmitoylcarnitine in fatty acid-degradation pathway. [file Image_8.PNG]

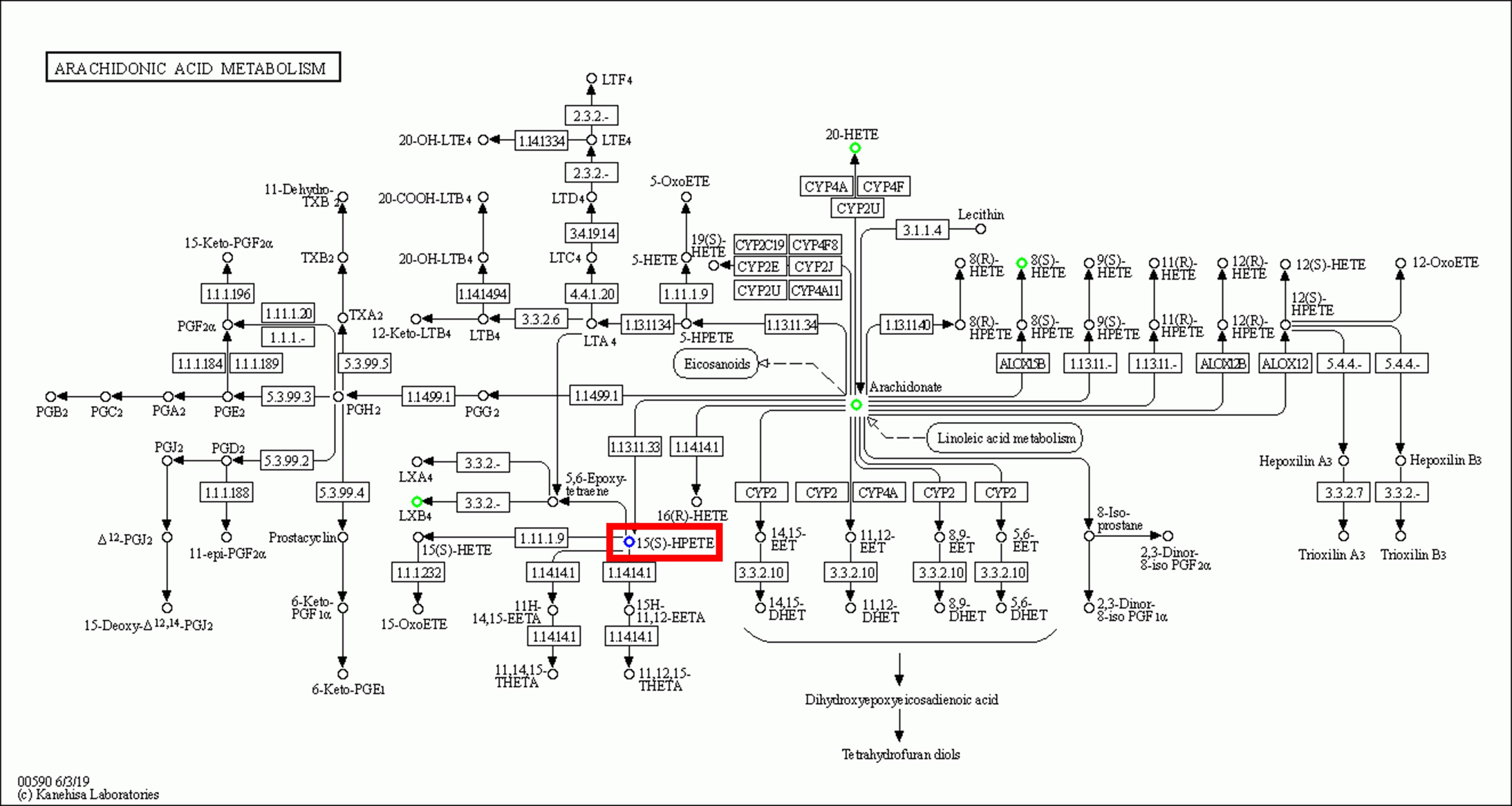

Supplement: Supplementary Figure 10 — 15(s)-hpete in an arachidonic-acid metabolic pathway. [file Image_10.PNG]

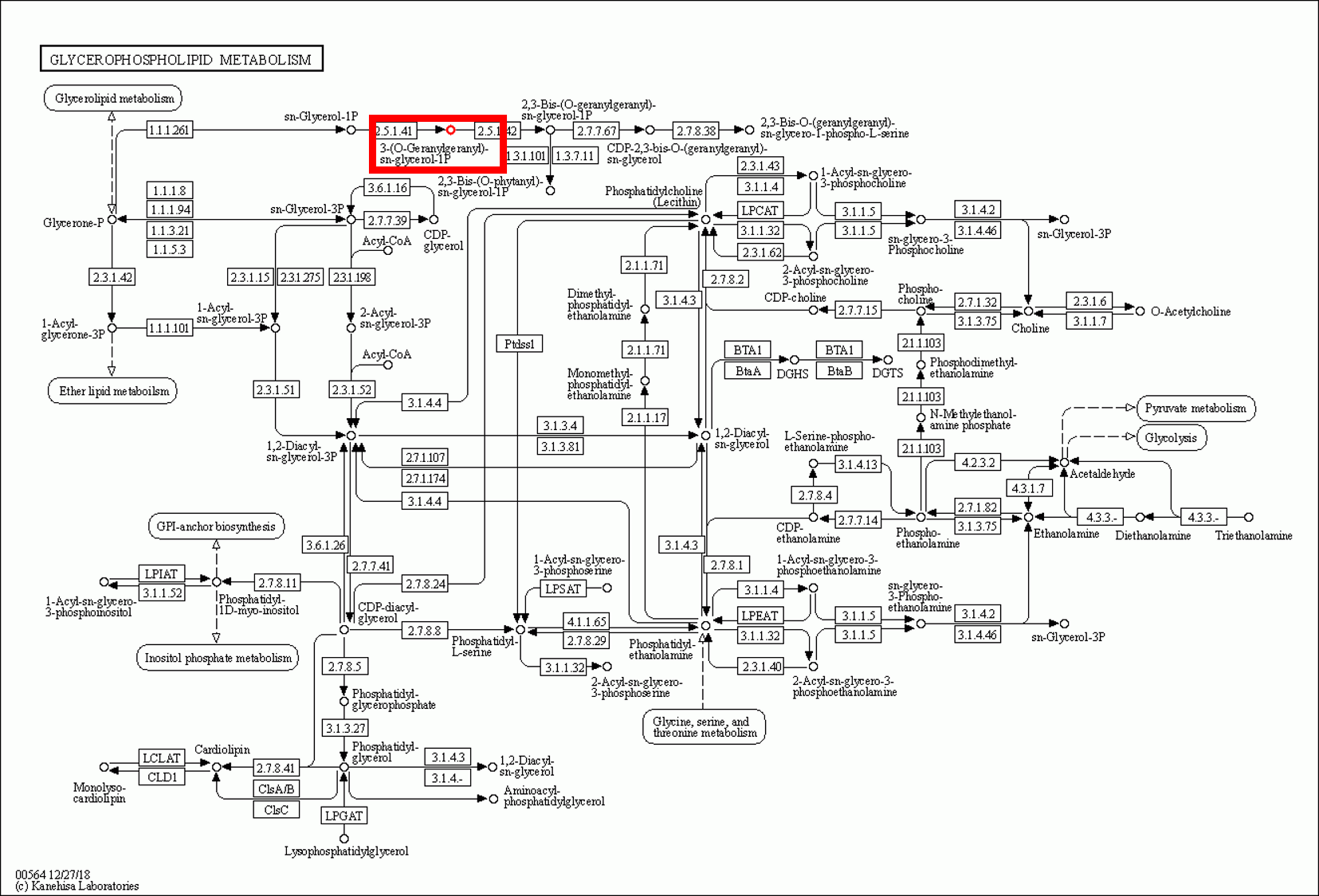

Supplement: Supplementary Figure 11 — Sn-3-o-(geranylgeranyl)glycerol 1-phosphate in a glycerophospholipid metabolic pathway. [file Image_11.PNG]

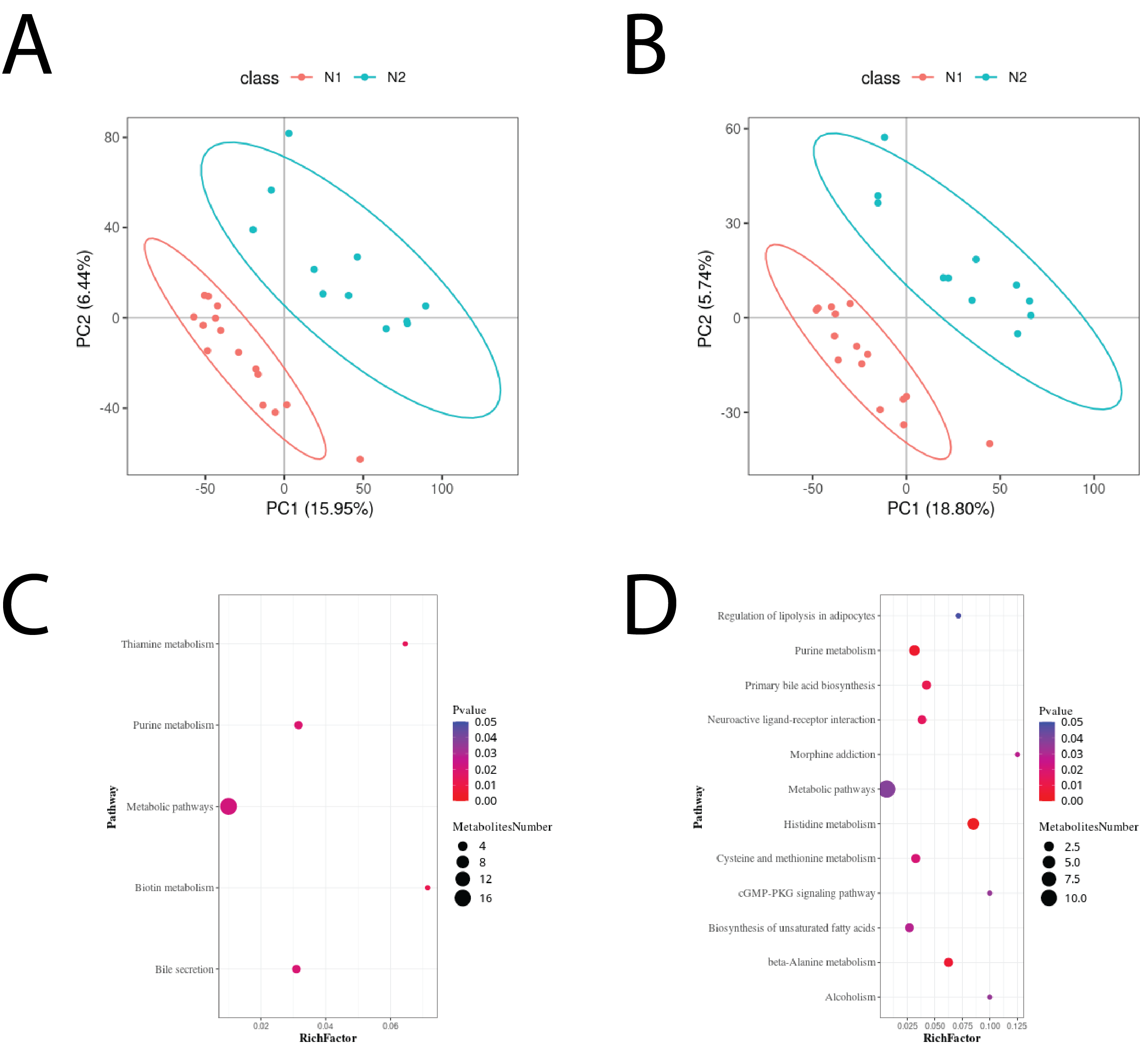

Supplement: Supplementary Figure 12 — Fecal metabolic profiles in the N2 group and N1 group. (A) PLS-DA of the two groups in ESI+ mode. (B) PLS-DA of the two groups in ESI–mode. (C) Enriched pathways of altered metabolic features in ESI+ mode according to the KEGG database. (D) Enriched pathways of altered metabolic features in ESI– mode according to the KEGG database. [file Image_12.PNG]

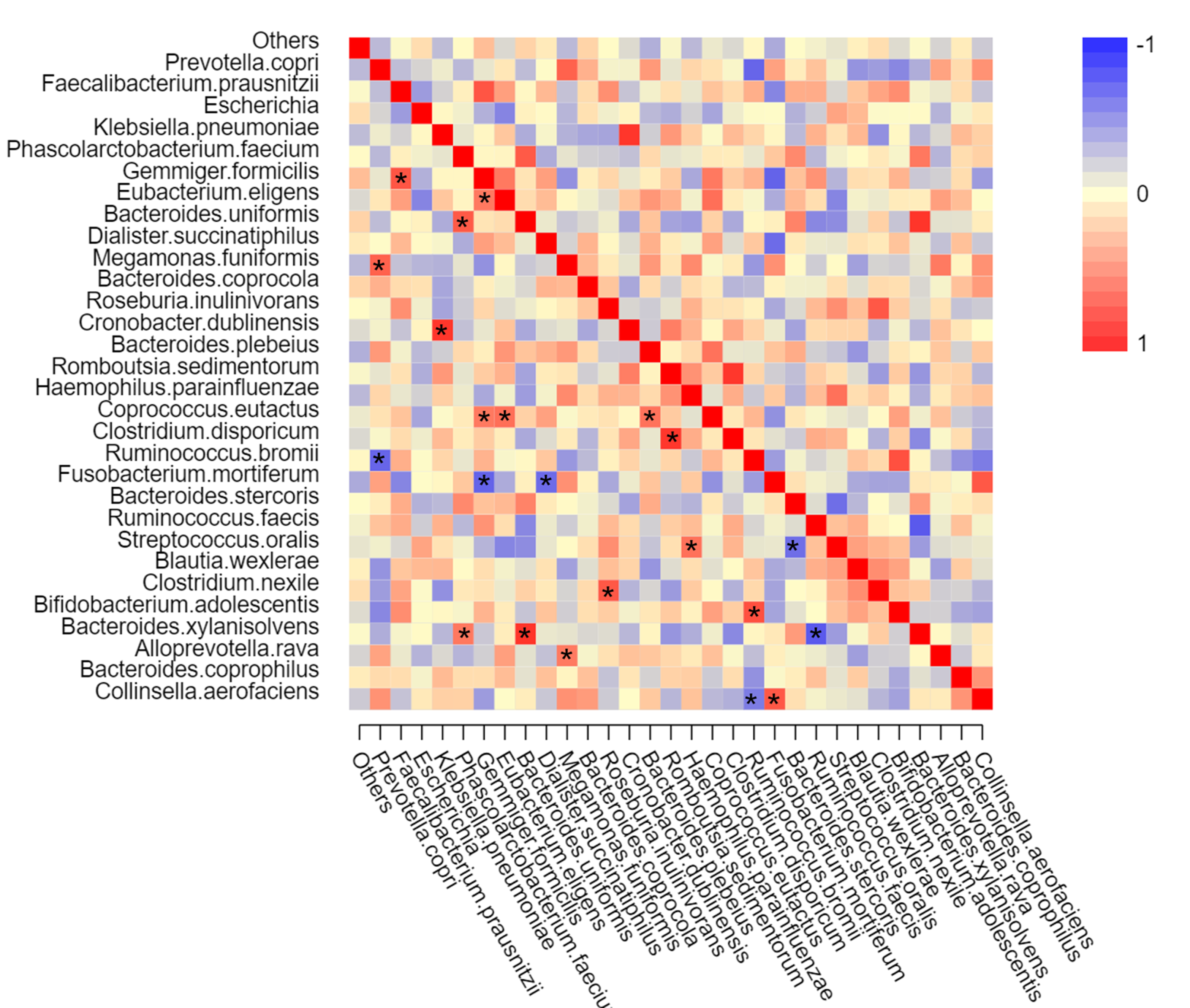

Supplement: Supplementary Figure 13 — The complicated network pattern between the distinct gut-microbiome profile. *Correlation coefficient >0.5 or < −0.5. [file Image_13.PNG]
